# Supplementary material for: FBXL7 Body Hypomethylation Is Frequent in Tumors from the Digestive and Respiratory Tracts and Is Associated with Risk-Factor Exposure
Source: Int J Mol Sci. 2022 Jul 15;23(14):7801. doi: 10.3390/ijms23147801 (PMC9316635; doi:10.3390/ijms23147801)
Supplement: Supplementary file 1 [file ijms-23-07801-s001.zip › Supplementary File 1.pdf]

Supplementary File 1: Summary of the patients included in the methylome analysis, by tumor type.

	**ESCC**	**LSCC**	**OCSCC**	**OPSCC**
Gender				
Male	21	18	15	8
Female	3	2	1	-
Age				
<60	14	6	11	3
≥60	7	14	4	5
Smoking history				
Yes	22	16	12	8
No	1	3	3	-
NA	1	1	-	-
Alcohol history				
Yes	20	7	11	8
No	3	7	4	-
NA	1	1	-	-
HPV status				
Positive	NA	NA	NA	5
Negative	NA	NA	NA	3

ESCC: esophageal squamous cell carcinoma; LSCC: laryngeal squamous cell carcinoma; OCSCC: oral cavity squamous cell carcinoma; OPSCC: oropharynx squamous cell carcinoma; NA: not available.
