# Supplementary material for: FBXL7 Body Hypomethylation Is Frequent in Tumors from the Digestive and Respiratory Tracts and Is Associated with Risk-Factor Exposure
Source: Int J Mol Sci. 2022 Jul 15;23(14):7801. doi: 10.3390/ijms23147801 (PMC9316635; doi:10.3390/ijms23147801)
Supplement: Supplementary file 1 [file ijms-23-07801-s001.zip › Supplementary File 3.pdf]

Supplementary File 2: Sensitivity, specificity, and accuracy values of each methylome probe mapping to *FBXL7* to distinguish non-tumor surrounding tissues ad esophageal squamous cell carcinoma.

Rank sorted by accuracy	Probe	Sensitivity	Specificity	Accuracy (AUC)
1	cg11339964	0.9583	0.9375	0.9818
2	cg04001359	0.9167	1.00	0.9661
3	cg24640610	0.9167	0.9375	0.9479
4	cg13394491	0.9167	0.9375	0.9453
5	cg09051630	0.8333	1.00	0.9453
6	cg24185447	0.8333	1.00	0.9297
7	cg25936482	0.9167	0.9375	0.9219
8	cg11005831	0.8333	0.9375	0.9193
9	cg18631301	0.9167	0.8125	0.9115
10	cg20328399	0.75	1.00	0.8932
11	cg26233084	0.75	1.00	0.8906
12	cg18620300	0.75	1.00	0.888
13	cg15540341	0.875	0.8125	0.8828
14	cg01572460	0.7917	0.9375	0.8672
15	cg13373080	0.6667	1.00	0.8568
16	cg00664723	0.75	0.8125	0.8542
17	cg13998890	0.7083	0.9375	0.8516
18	cg20771687	0.7917	1.00	0.8464
19	cg18373623	0.8333	0.8125	0.8411
20	cg02519948	0.8333	0.8125	0.8411
21	cg20011459	0.6667	0.875	0.8333
22	cg19641327	0.7917	0.875	0.8255
23	cg06055044	0.8333	0.6875	0.8177
24	cg08792955	0.7083	0.8125	0.8047
25	cg23041295	0.4583	1.00	0.7891
26	cg18202928	0.75	0.875	0.7813
27	cg07238450	0.9167	0.5625	0.7734
28	cg01149929	0.5833	1.00	0.7734
29	cg25623727	0.8333	0.6875	0.7656
30	cg00960263	0.5	1.00	0.763
31	cg05023415	0.5833	1.00	0.7578
32	cg07856071	0.6667	0.9375	0.7474
33	cg14667871	1	0.375	0.7161
34	cg16679007	0.7917	0.6875	0.7083
35	cg16641358	0.6667	0.9375	0.7083
36	cg10047595	1	0.375	0.7031
37	cg19632594	0.7917	0.625	0.6979
38	cg02704181	0.5833	0.8125	0.6849
39	cg06577205	0.4167	0.9375	0.6563
40	cg24872782	0.75	0.625	0.6432
41	cg15108430	0.4167	0.9375	0.638
42	cg26134895	0.5833	0.75	0.6198
43	cg00219774	0.4167	0.875	0.5755
44	cg06468166	0.2917	0.9375	0.5599
45	cg08849558	0.9167	0.50	0.5521
46	cg14583673	0.4167	0.6875	0.5156
47	cg08235984	0.7083	0.4375	0.513
48	cg12910969	0.6667	0.50	0.4948
