# Supplementary figures and images for: FBXL7 Body Hypomethylation Is Frequent in Tumors from the Digestive and Respiratory Tracts and Is Associated with Risk-Factor Exposure
Source: Int J Mol Sci. 2022 Jul 15;23(14):7801. doi: 10.3390/ijms23147801 (PMC9316635; doi:10.3390/ijms23147801)

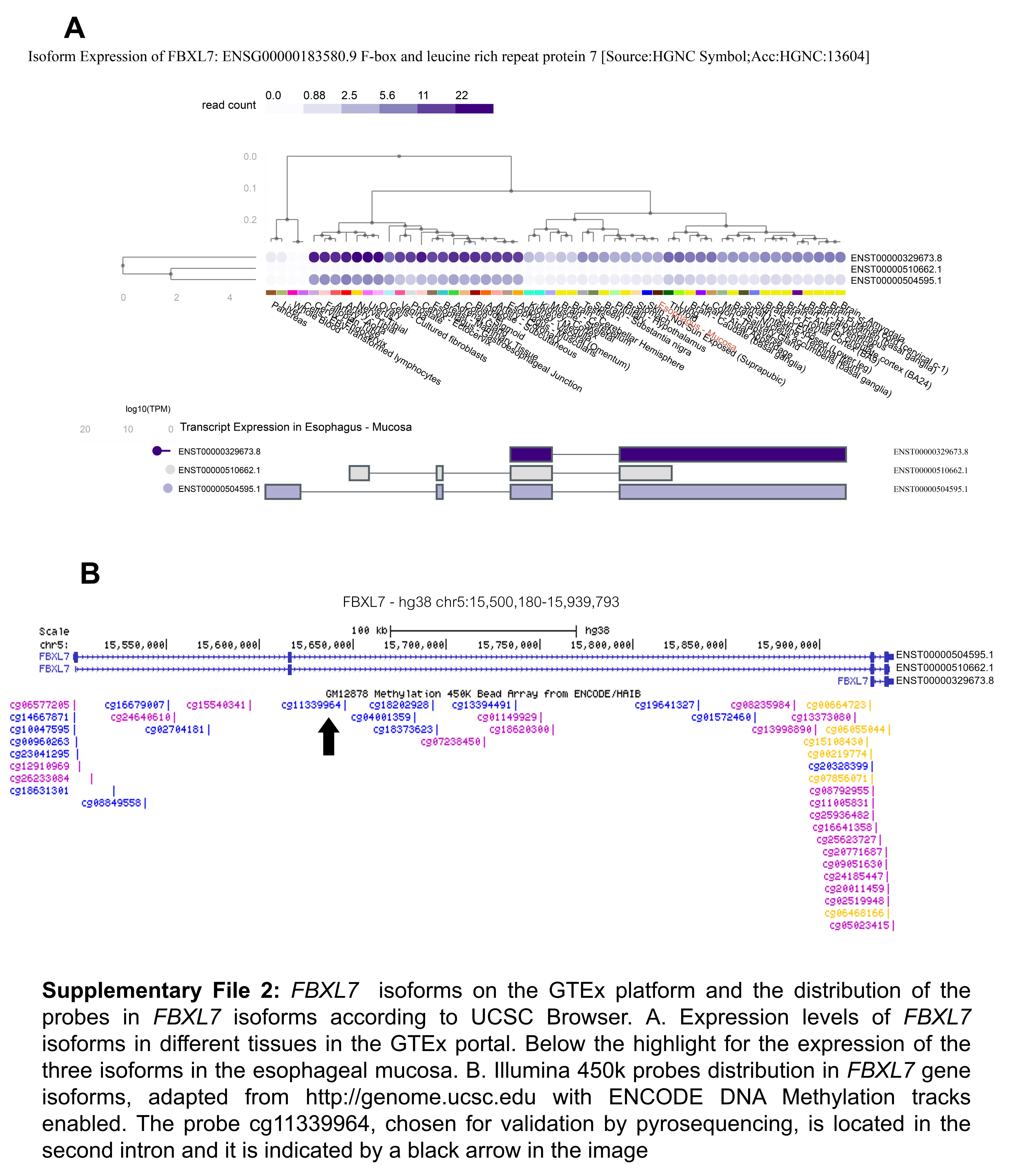

Supplement: Supplementary file 1 [file ijms-23-07801-s001.zip › Supplementary File 2.jpg]

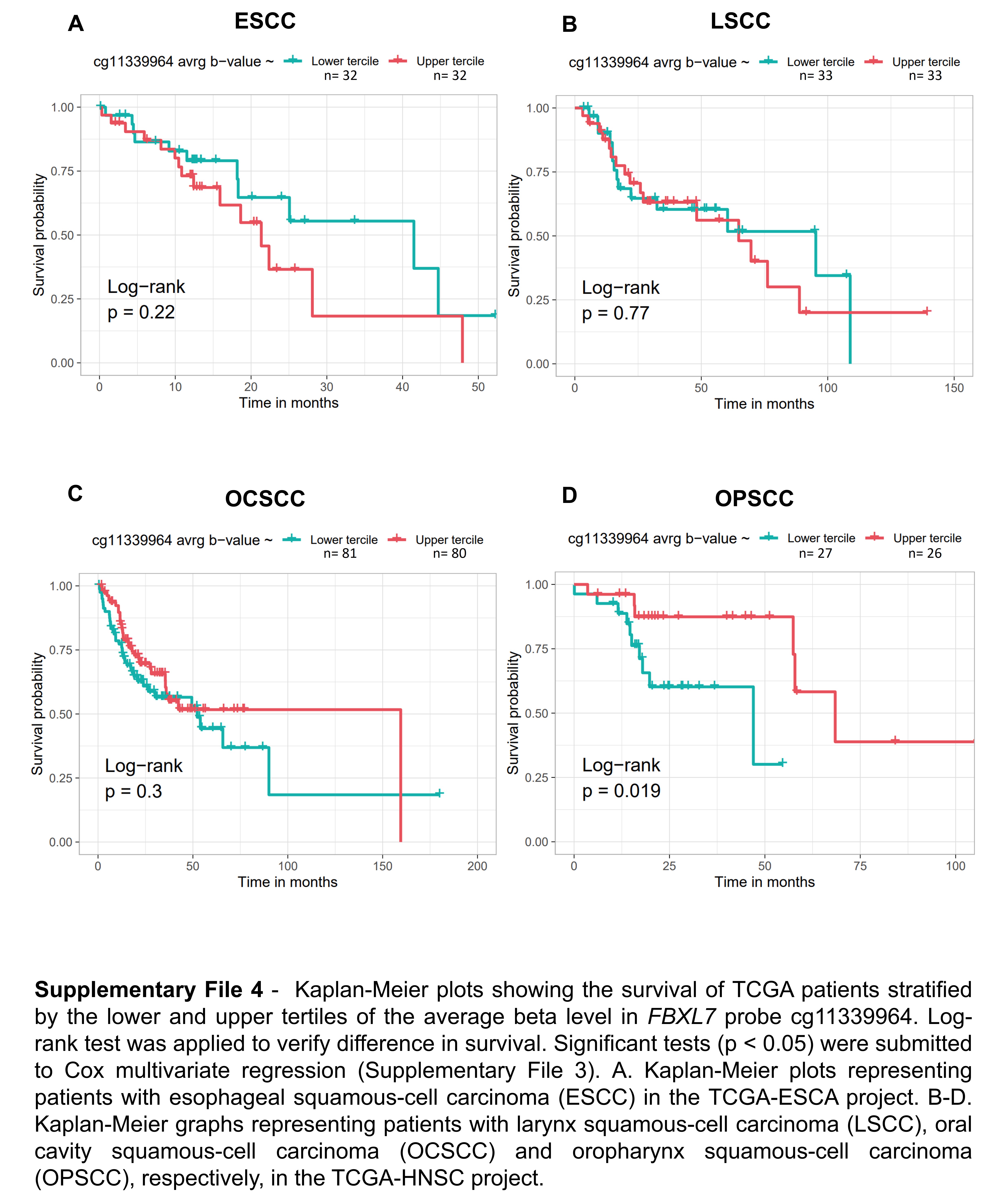

Supplement: Supplementary file 1 [file ijms-23-07801-s001.zip › Supplementary File 5.jpg]

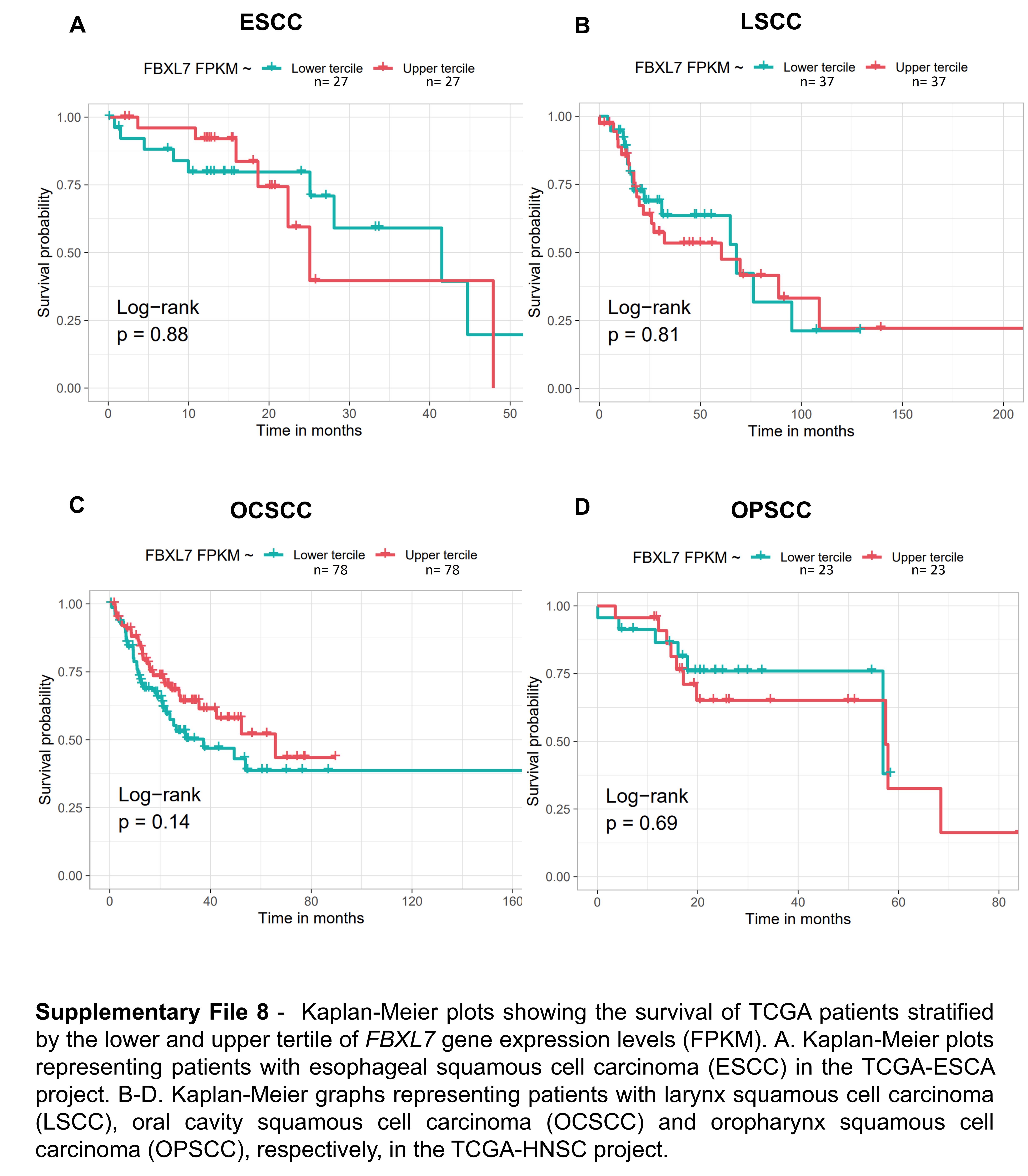

Supplement: Supplementary file 1 [file ijms-23-07801-s001.zip › Supplementary File 8.jpg]
